# Associations of cholesterol, high-density lipoprotein and glucose index and triglyceride–glucose-related indices with carotid atherosclerosis in young and middle-aged individuals

**DOI:** 10.3389/fendo.2026.1771118

**Published:** 2026-03-17

**Authors:** Kexin Du, Yiping Zhang, Xuefei Zhang, Yuxin Pan, Tao Pan, Yuxuan Tong, Xiao Chen, Dongling Lv

**Affiliations:** 1 Department of Cardiology, Affiliated Hospital of Nanjing University of Chinese Medicine, Nanjing, China; 2 Department of Radiology, Affiliated Hospital of Nanjing University of Chinese Medicine, Nanjing, China; 3 Department of General Practice, the Drum Tower Hospital Affiliated to the Medical School of Nanjing University, Nanjing, China

**Keywords:** glucose index, carotid atherosclerosis, cholesterol, high-density lipoprotein, triglyceride-glucose, young and middle-aged population

## Abstract

**Background:**

Associations of cholesterol, high-density lipoprotein, and glucose (CHG) index and triglyceride-glucose (TYG)-related indices are biomarkers of insulin resistance. However, the relationships between carotid atherosclerosis and the CHG index and TYG-related indices in young and middle-aged people are unclear. This cross-sectional study reported such associations in a general population.

**Methods:**

A total of 9,110 participants aged between 18 and 60 years were included. TYG index (ln(triglyceride (mg/dL) × blood glucose (mg/dL)/2), TYG-BMI index (TYG×BMI), TYG/HDL index (TYG/HDL cholesterol), and CHG index (ln[cholesterol (mg/dL) × blood glucose (mg/dL)/2 × HDL (mg/dL)) were calculated. The associations between carotid atherosclerosis and CHG- and TYG-related indices were analyzed via logistic regression analyses.

**Results:**

Carotid atherosclerosis was observed in 3,089 (33.9%) participants. CHG index was significantly associated with carotid atherosclerosis (odds ratio (OR): 2.47, 95% confidence interval (CI): 1.87–3.26; Q4 vs. Q1, OR: 1.79, 95% CI: 1.42–2.26). Similar associations were observed for the TYG index (OR: 1.26, 95% CI: CI: 1.08–1.48; Q4 vs. Q1, OR: OR: 1.32, 95% CI: 1.08–1.62), TY-BMI (OR: 1.01, 95% CI: 1.00–1.02; Q4 vs. Q1, OR: 1.55, 95% CI: 1.15–2.11) and TYG/HDL (OR: 1.10, 95% CI: 1.06–1.13; Q4 vs. Q1, OR: 1.60, 95% CI: 1.32–1.94). CHG had better performance than the TYG, TYGBMI, and TYGHDL in identifying carotid atherosclerosis (the area under the curve was 0.642 vs 0.599, 0.620, and 0.612, respectively).

**Conclusion:**

CHG index and TYG-related indices were associated with carotid atherosclerosis in young and middle-aged individuals. CHG had better performance than did TYG-related indices in identifying carotid atherosclerosis.

## Introduction

Cardiovascular diseases (CVDs) are the leading cause of death globally, with a continuously increasing disease burden (1). The Global Burden of Disease Study from 1990–2019 revealed that the prevalence of CVD increased by 92.3%, with the number of deaths increasing from 12.1 million to 18.6 million, accounting for one-third of all-cause mortality (1, 2). As a large developing country with an accelerating aging population, China has a significantly higher age-standardized incidence rate of CVD than the global population does (3). Atherosclerosis serves as a critical pathological foundation for cardiovascular diseases (4). Therefore, early identification and intervention of the core mechanism—atherosclerosis—become the key to prevention and control.

Insulin resistance (IR) is a key driver of CVD development, but its clinical assessment relies on complex gold-standard techniques such as the hyperinsulinemic-euglycemic clamp, which are impractical for widespread use (5, 6). The triglyceride-glucose (TYG) index, a biomarker of IR, has been validated in multiple cohort studies as an effective tool for predicting CVD risk (7, 8). Recently, composite metabolic biomarkers, which integrate multidimensional pathophysiological information, have gained attention. For example, the TYG-body mass index (BMI) enhances CVD predictive power by incorporating obesity (3, 9, 10). The cholesterol-high-density lipoprotein-glucose (CHG) index is another marker closely related to IR, metabolic syndrome, and diabetes (11, 12). A recent study revealed that CHG is associated with CVD risk and has similar predictive value to TYG (12). However, this study lacked a specific analysis of the young and middle-aged population (40–65 years) and did not systematically compare the predictive efficacy of CHG with that of other TYG-derived indices, such as TYG-BMI and TYG/HDL, in carotid atherosclerosis (12).

In middle-aged and young people, the insidious accumulation of metabolic abnormalities is a key driver of subclinical atherosclerosis. Even cardiovascular risk factors within the normal range are associated with subclinical atherosclerosis and long-term CVD events in young individuals (13). Recent studies have shown increasing CVD incidence in younger and middle-aged populations (14–18), with increasing hospitalization rates for cardiovascular diseases and acute stroke (15–17). The carotid artery acts as a “window” for systemic atherosclerosis. Carotid atherosclerosis or plaque formation significantly increases the risk of adverse outcomes such as stroke, which is a major cause of premature death (19, 20) and is widely used as a predictor of individual cardiovascular events (19, 21). One-quarter of Chinese adults have increased carotid intima–media thickness or carotid plaque (22). This study is the first to explore the relationship between the CHG index and carotid atherosclerosis in middle-aged and young people and to analyze its differences from TYG-related indices in predicting carotid atherosclerosis, providing a novel stratification tool for the primary prevention of metabolism-related CVD.

## Methods

### Data source and study population

**Figure 1:**
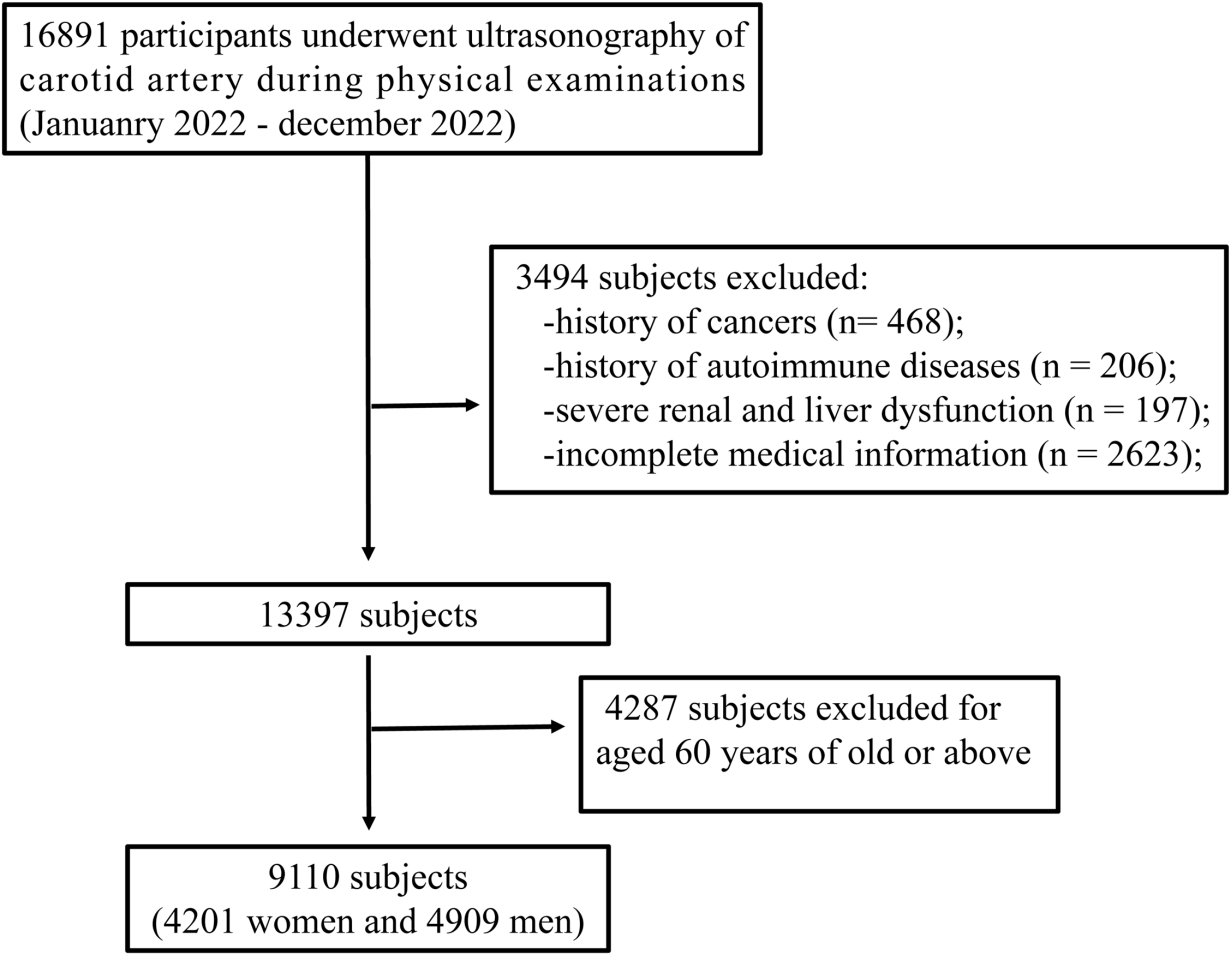
Flowchart of participant inclusion.

The retrospective cross-sectional study data were obtained from the physical examination database of Gulou Hospital and the Affiliated Hospital of Nanjing University of Chinese Medicine in Jiangsu Province in 2021. A total of 9,110 participants aged between 18 and 60 years (with a mean age of 48.84 ± 8.18 years) were included. The exclusion criteria were as follows: having serious chronic diseases (except for hypertension and diabetes mellitus, which were specifically mentioned in the study), having a recent history of malignant tumor, major surgery or trauma, and being younger than 18 years or older than 60 years. Flowchart of participant inclusion is shown in **Figure 1**. The study conformed to the medical ethics code and was reviewed and approved by the Ethics Committee of Affiliated Hospital of Nanjing University of Chinese Medicine.

### Dada collection and assessment of TYG and CHG indices

Age, sex, body weight, height, history of diabetes and hypertension, uric acid, albumin, liver (aspartate aminotransferase, AST; alanine aminotransferase, ALT) and renal function data were collected from the medical system. The levels of triglycerides, total cholesterol, high-density lipoprotein cholesterol (HDL-C), high-density lipoprotein cholesterol (LDL-C), and blood glucose were measured on the same day at the two institutions via an automatic biochemical analyzer. Body mass index (BMI) is calculated by dividing a person’s weight in kilograms by the square of their height in meters. The formula for calculating the TYG index is as follows: TYG Index = ln(TG (mg/dL) × blood glucose (mg/dL)/2). The TYG-BMI index is the product of TYG multiplied by BMI. The TYG/HDL index is the ratio of TYG to HDL cholesterol. The formula for calculating the CHG index is as follows: CHG index = ln[TC (mg/dL) × blood glucose (mg/dL)/2 × HDL (mg/dL)]. CHG and TYG were divided into four groups (Q1-Q4) on the basis of quartiles. The CHG-TYG scores were subsequently calculated. For example, if a person had Q1 for CHG and Q1 for TYG, the CHG-TYG score would be two.

### Evaluation of carotid atherosclerosis

The occurrence of carotid atherosclerosis was the primary endpoint event. Carotid arteries were evaluated by high-resolution ultrasound to measure indicators such as carotid intima–media thickness. Carotid atherosclerosis was diagnosed when the IMT was ≥1.0 mm or when localized plaque formation (Carotid intimal thickening and plaque formation) was detected in the carotid arteries. The ultrasound examination was carried out by a specialized ultrasonographer to ensure the accuracy and consistency of the results.

### Clinical trial number

Not applicable.

### Statistical analysis

The data were analyzed via SPSS 29.0. Descriptive statistics were employed to summarize the characteristics of the participants. The quantitative measurement data are expressed as the means ± standard deviations, and the count data are expressed as frequencies and percentages (n, %). Differences in measurement data between the carotid atherosclerosis and noncarotid atherosclerosis groups were compared via independent-samples t tests or Mann–Whitney U tests, whereas differences in count data were compared via the chi–square test. Multivariable logistic regression models were constructed to analyze the associations between CHG and TYG-related indices and the risk of carotid atherosclerosis: Model 1, adjusted for age, sex, and BMI; Model 2, Model 1 + liver function (ALT, AST), renal function, diabetes status, albumin, uric acid, and blood pressure; Model 3: Model 2 + lipid parameters (LDL-C, TG, HDL-C).

The dose–response relationship between each index and the risk of carotid atherosclerosis was further explored via restricted cubic spline analysis to better visualize the nonlinear relationship between them. Subgroup analyses stratified by sex, hypertension status, LDL-C level, and diabetes status were conducted to evaluate the differences in the associations of each index in different subgroups and to determine whether there were differences in the relationships between these indices and carotid atherosclerosis risk in different populations. Receiver operating characteristic (ROC) curves were plotted, the area under the curve (AUC) was calculated, and the predictive abilities of the TYG index, TYGBMI, TYG-HDL ratio, and CHG were compared. A p value less than 0.05 represents statistical significance.

## Results

### Characteristics of the subjects

**Figure 2:**
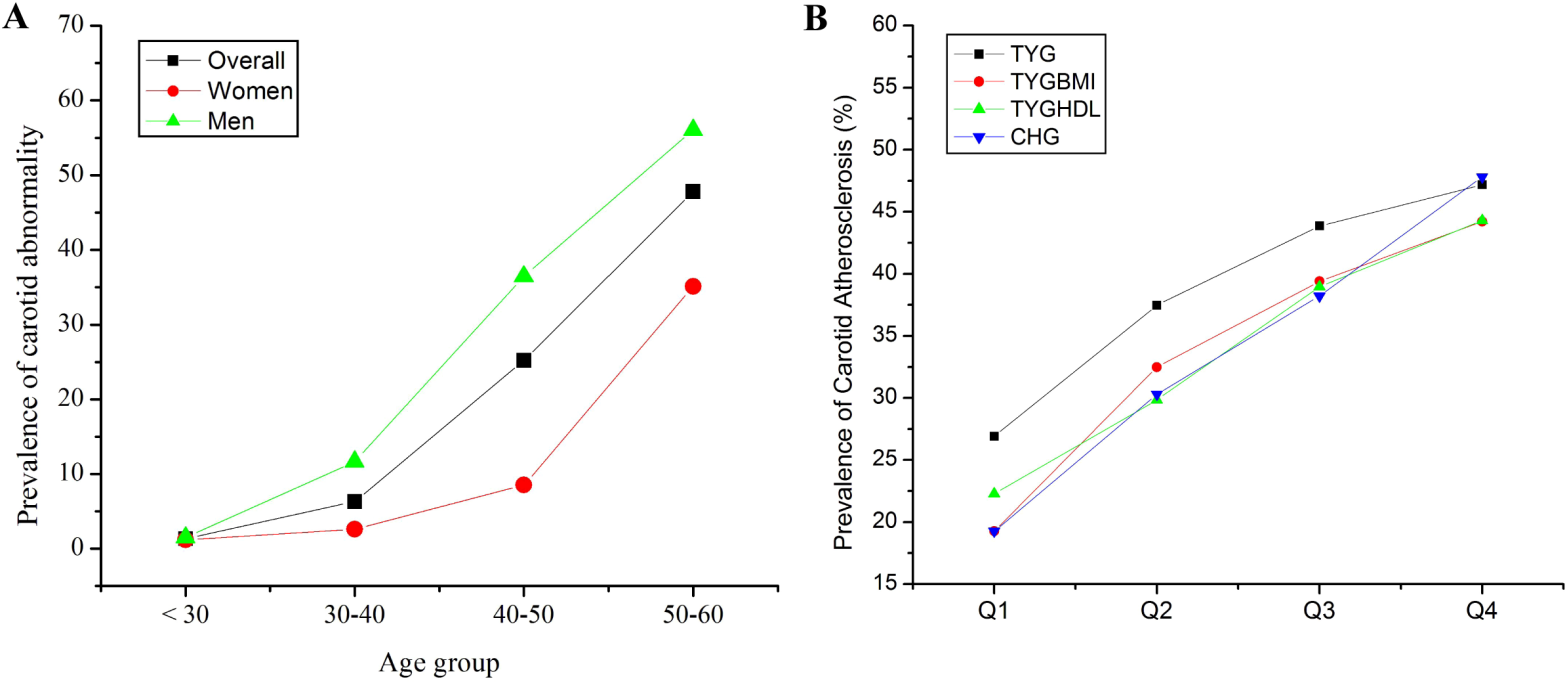
Prevalence of carotid atherosclerosis in different age groups **(A)** and in different IQRs of the triglyceride glucose (TYG) index, TYG-body mass index (TYGBMI), TYG/high-density lipoprotein cholesterol (TYGHDL) ratio, and cholesterol, high-density lipoprotein, and glucose index (CHG) **(B)**.

The study included 9,110 participants (53.89% male, mean age 48.84 ± 8.18 years), with 3,089 (33.9%) diagnosed with carotid atherosclerosis (**Table 1**). Significant differences (p < 0.001) were observed between the groups: the atherosclerosis group was older (52.57 ± 5.27 vs. 45.61 ± 9.01 years), had a higher male prevalence (71.34% vs. 44.92%), had an elevated BMI (25.01 ± 3.05 vs. 24.17 ± 3.37 kg/m²), and had worse metabolic profiles, including higher LDL-c (3.08 ± 0.85 vs. 2.93 ± 0.76 mmol/L), blood glucose (5.54 ± 1.37 vs. 5.22 ± 1.06 mmol/L), and blood pressure (SBP: 130.00 ± 15.31 vs. 126.7 ± 16.35 mmHg). Hypertension (30.56% vs. 14.65%) and diabetes (8.97% vs. 3.5%) were more prevalent in the atherosclerosis group (p < 0.001). The atherosclerosis group also presented higher levels of TYG, TYG-BMI, TYGHDL and CHG (P < 0.001). Men had a greater incidence of carotid atherosclerosis than women did (**Figure 2A**). The prevalence of carotid atherosclerosis increased with increasing TYG, TYG-BMI, TYGHDL and CHG (**Figure 2B**).

**Table 1:** Characteristics of the subjects divided by carotid atherosclerosis status.

Variables		Carotid atherosclerosis		P
	Overall (n = 9110)	Yes (n = 3089)	No (n = 6021)	
Age (years)	48.84 ± 8.18	52.57 ± 5.27	45.61 ± 9.01	**< 0.001**
Sex (men)	4909 (53.89%)	2204 (71.34%)	2705 (44.92%)	**< 0.001**
Weight (kg)	67.60 ± 12.34	80.00 ± 11.78	66.98 ± 12.85	**< 0.001**
Height (cm)	166.32 ± 8.09	168.08 ± 7.80	165.97 ± 8.13	**< 0.001**
BMI (kg/m^2^)	24.31 ± 3.22	25.01 ± 3.05	24.17 ± 3.37	**< 0.001**
AST (U/L)	21.25 ± 9.53	21.89 ± 10.57	20.97 ± 8.90	**< 0.001**
ALT (U/L)	23.46 ± 19.01	24.73 ± 17.53	22.89 ± 19.79	**< 0.001**
Albumin (mmol/L)	44.68 ± 1.76	44.59 ± 1.77	44.73 ± 1.75	**0.001**
Uric acid (μmol/L)	334.58 ± 84.68	350.84 ± 82.31	326.50 ± 85.05	**< 0.001**
Creatinine (mmol/L)	65.34 ± 17.45	68.61 ± 14.71	63.60 ± 18.33	**< 0.001**
Blood glucose (mmol/L)	5.33 ± 1.16	5.54 ± 1.37	5.22 ± 1.06	**< 0.001**
HDL-c (mmol/L)	1.40 ± 0.38	1.33 ± 0.36	1.44 ± 0.38	**< 0.001**
LDL-c (mmol/L)	2.98 ± 0.76	3.08 ± 0.85	2.93 ± 0.76	**< 0.001**
TC (mmol/L)	5.10 ± 0.96	5.19 ± 1.00	5.06 ± 0.93	**< 0.001**
TG (mmol/L)	1.46 ± 1.43	1.57 ± 1.19	1.42 ± 1.44	**< 0.001**
SBP (mmHg)	127.81 ± 16.23	130.00 ± 15.31	126.7 ± 16.35	**< 0.001**
DBP (mmHg)	76.56 ± 13.09	77.90 ± 12.61	75.86 ± 13.08	**< 0.001**
Hypertension	1862 (18.79%)	944 (30.56%)	882 (14.65%)	**<0.001**
Diabetes	489 (5.37%)	277 (8.97%)	212 (3.5%)	**< 0.001**
CKD	30 (0.33%)	15 (0.5%)	15 (0.3%)	0.06
CHG	6.76 ± 0.35	6.87 ± 0.35	6.70 ± 0.33	**< 0.001**
TYG	8.52 ± 0.64	8.65 ± 0.62	8.47 ± 0.64	**< 0.001**
TYG-BMI	207.49 ± 32.80	216.53 ± 31.12	204.66 ± 33.35	**< 0.001**
TYG/HDL-c	6.55 ± 1.90	7.01 ± 1.93	6.31 ± 1.85	**< 0.001**

AST, aspartate aminotransferase; BMI, body mass index; CHG, cholesterol, high-density lipoprotein, and glucose index; CKD, chronic kidney disease; DBP, diastolic blood pressure; HDL-c, high-density lipoprotein cholesterol; LDL-c, low-density lipoprotein cholesterol; SBP, systolic blood pressure; TC, total cholesterol; TG, triglyceride; TYG, triglyceride glucose. Bold values mean p<0.05.

### Associations of CHG and TYG-related indices with carotid atherosclerosis

**Table 2** presents the associations between CHG and TYG-related indices and the risk of carotid atherosclerosis. The CHG index, both as a continuous variable and across quartiles, demonstrated a significant association with carotid atherosclerosis across all the models. According to the fully adjusted model, a per-unit increase in CHG was associated with a 2.47-fold greater risk (odds ratio (OR) = 2.47, 95% confidence interval (CI): 1.87–3.26). Compared with those in the lowest quartile (Q1), individuals in Q4 presented a significantly increased risk (OR = 1.79, 95% CI: 1.42–2.26, p < 0.001).

**Table 2:** Association between CHG or TYG-related indices and the risk of carotid atherosclerosis.

Variables	Model 1	p	Model 2	p	Model 3	p
	OR (95%CI)		OR (95%CI)		OR (95%CI)	
CHG (continuous)	2.29 (1.97-2.67)	**< 0.001**	1.28 (1.89-2.76)	**< 0.001**	2.47 (1.87-3.26)	**< 0.001**
Q1 (< 6.51)	1		1		1	
Q2 (6.51-6.74)	1.22 (1.07-1.45)	**0.004**	1.22 (1.04-1.43)	**0.03**	1.19 (1.00-1.42)	0.06
Q3 (6.74-6.98)	1.55 (1.33-1.80)	**< 0.001**	1.58 (1.34-1.86)	**< 0.001**	1.50 (1.24-1.82)	**< 0.001**
Q4 (> 6.98)	2.01 (1.72-2.34)	**< 0.001**	1.91 (1.60-2.27)	**< 0.001**	1.79 (1.42-2.26)	**< 0.001**
TYG (continuous)	1.31 (1.22-1.42)	**< 0.001**	1.19 (1.07 -1.33)	**0.002**	1.26 1.08 -1.48)	**0.006**
Q1 (<8.08)	1		1		1	
Q2 (8.08-8.48)	1.27 (1.10-1.47)	**0.001**	1.21 (1.03-1.41)	**0.018**	1.20 (1.02-1.40)	**0.03**
Q3 (8.48-8.92)	1.52 (1.32-1.75)	**<0.001**	1.42 (1.21-1.66)	**< 0.001**	1.41 (1.20-1.66)	**< 0.001**
Q4 (> 8.92)	1.62 (1.40-1.86)	**< 0.001**	1.34 (1.12-1.60)	**0.001**	1.32 (1.08-1.62)	**0.006**
TYG/HDL (continuous)	1.07 (1.04-1.10)	**< 0.001**	1.08 (1.05 -1.12)	**< 0.001**	1.10 (106 -1.13)	**< 0.001**
Q1 (< 5.13)	1		1		1	
Q2 (5.13-6.33)	1.09 (0.94-1.27)	0.23	1.14 (0.97-1.35)	0.11	1.16 (0.98-1.38)	0.09
Q3 (6.33-7.70)	1.31 (1.13-1.53)	**< 0.001**	1.40 (1.18-1.66)	**< 0.001**	1.44 (1.20-1.73)	**< 0.001**
Q4 (> 7.70)	1.38 (1.17-1.61)	**< 0.001**	1.50 (1.25-1.79)	**< 0.001**	1.60 (1.32-1.94)	**< 0.001**
TYG-BMI (continuous)	1.01 (1.01-1.01)	**< 0.001**	1.01 (1.00 -1.01)	**0.002**	1.01 (1.00 -1.02)	**0.004**
Q1 (< 184.4)	1		1		1	
Q2 (184.4-205.2)	1.40 (1.19-1.64)	**< 0.001**	1.24 (1.03-1.48)	**0.02**	1.20 (1.00-1.44)	0.06
Q3 (205.2-227.5)	1.61 (1.34-1.93)	**< 0.001**	1.40 (1.14-1.73)	**0.001**	1.35 (1.09-1.68)	**0.007**
Q4 (> 227.5)	2.05 (1.63-2.56)	**< 0.001**	1.59 (1.21-2.10)	**0.001**	1.55(1.15-2.11)	**0.005**

Model 1 was adjusted for age, sex and body mass index; Model 2 was further adjusted for liver function, renal function, diabetes, albumin, uric acid, and blood pressure. Model 3 was further adjusted for low-density lipoprotein cholesterol, triglyceride or high-density lipoprotein cholesterol (for TYG indices).

TYG, triglyceride glucose; TYG-BMI, TYG*body mass index; TYG/HDL, TYG/high-density lipoprotein cholesterol (C); CHG, cholesterol, high-density lipoprotein, and glucose index.

CI, confidence interval; HDL-c, high-density lipoprotein cholesterol; OR, odds ratio; TYG, triglyceride glucose.Bold values mean p<0.05.

Similarly, the TYG index was significantly associated with carotid atherosclerosis. According to the fully adjusted model, the continuous TYG index remained a significant associated factor for carotid atherosclerosis (OR = 1.26, 95% CI: 1.08–1.48). A dose–response relationship was observed across quartiles, with Q4 showing a 1.32-fold increased risk compared with that of Q1 (OR = 1.32, 95% CI: 1.08–1.62).

**Figure 3:**
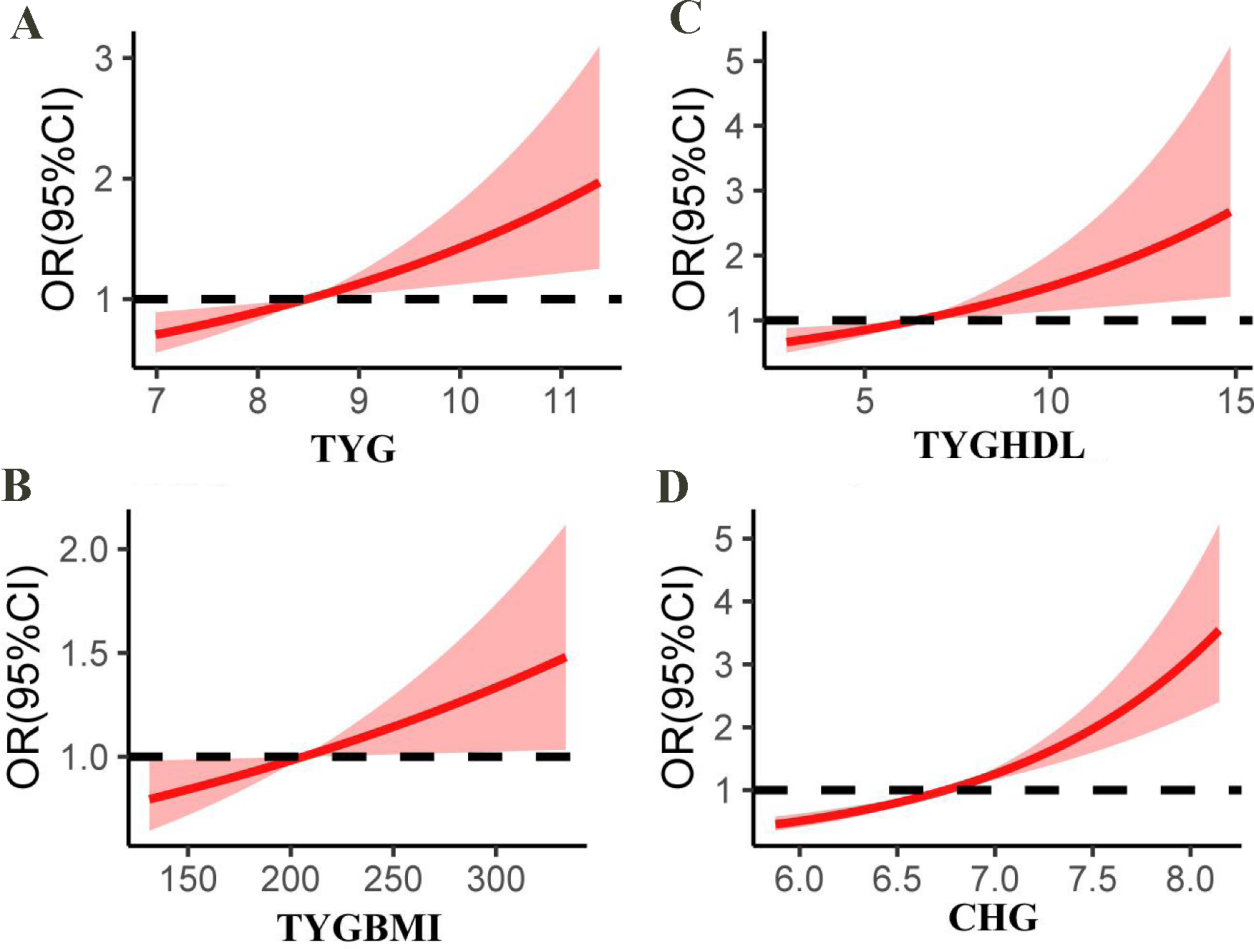
Restricted cubic splines show the multivariable adjusted odds ratio for the risk of carotid atherosclerosis according to the triglyceride glucose (TYG) index **(A)**, TYG-body mass index (TYGBMI) **(B)**, TYG/high-density lipoprotein cholesterol (TYGHDL) ratio **(C)**, and cholesterol, high-density lipoprotein, and glucose index (CHG) **(D)**. Age, sex, BMI, liver function, renal function, albumin, diabetes, uric acid, blood pressure, low-density lipoprotein cholesterol, total cholesterol (not for CHG), high-density lipoprotein cholesterol (for TYG and TYGBMI) or triglycerides (for CHG) were adjusted.

The TYG/HDL index was also significantly associated with carotid atherosclerosis. In Model 3, the continuous variables were positively correlated (OR = 1.10, 95% CI: 1.06–1.13). Notably, participants in Q4 had a 1.60-fold increased risk compared with those in Q1 (OR = 1.60, 95% CI: 1.32–1.94). The TYG-BMI index was significantly associated with carotid atherosclerosis in all the models. In Model 3, the continuous variable remained significant (OR = 1.01, 95% CI: 1.00–1.02). Compared with Q1, Q4 presented a 1.55-fold increased risk (OR = 1.55, 95% CI: 1.15–2.11). Intermediate quartiles (Q2 and Q3) showed progressively elevated risks, highlighting a dose–dependent relationship between higher levels of CHG, TYG, TYG/HDL, and TYG-BMI indices and carotid atherosclerosis risk. The restricted cubic splines analysis further revealed a positive dose–response relationship linking CHG, TYG-related indices, and carotid atherosclerosis risk (**Figure 3**).

We further explored the associations between CHG-TYG phenotypes and the risk of carotid atherosclerosis (**Table 3**). Compared with the lowest CHG-TYG score (< 2), the risk of carotid atherosclerosis significantly increased, especially for CHG-TYG scores > 5 (score = 5, OR = 1.61, 95% CI: 1.21–2.13; score = 6, OR = 1.62, 95% CI: 1.20–2.19; score = 7, OR: 1.92, 95% CI: 1.39–2.65; score = 8, OR = 1.83, 95% CI: 1.25–2.67).

**Table 3:** Association between the CHG and TYG phenotypes and the risk of carotid atherosclerosis.

Variables	Model 1	p	Model 2	p	Model 3	p
	OR (95%CI)		OR (95%CI)		OR (95%CI)	
CHG-TYG score						
2	1		1		1	
3	1.36 (1.05-1.78)	**0.02**	1.21 (0.91-1.61)	0.19	1.15 (0.86-1.53)	0.36
4	1.66 (1.30-2.13)	**< 0.001**	1.52 (1.17-1.99)	**< 0.001**	1.40 (1.06-1.85)	**0.02**
5	1.97 (1.55-2.51)	**< 0.001**	1.79 (1.38-2.33)	**< 0.001**	1.61 (1.21-2.13)	**0.001**
6	2.09 (1.63-2.67)	**< 0.001**	1.87 (1.43 -2.44)	**< 0.001**	1.62 (1.20-2.19)	**0.002**
7	2.57 (2.00-3.32)	**<0.001**	2.26 (1.71-2.98)	**< 0.001**	1.92 (1.39-2.65)	**<0.001**
8	2.99 (2.30-3.90)	**< 0.001**	2.26 (1.67-3.05)	**< 0.001**	1.83 (1.25-2.67)	**0.002**

Model 1 was adjusted for age, sex and body mass index; Model 2 was further adjusted for liver function, renal function, diabetes, albumin, uric acid, and blood pressure. Model 3 was further adjusted for low-density lipoprotein cholesterol, triglyceride, cholesterol, and high-density lipoprotein cholesterol.

TYG, triglyceride glucose; CHG, cholesterol, high-density lipoprotein, and glucose index.

CI, confidence interval; OR, odds ratio; TYG, triglyceride glucose. Bold values mean p<0.05.

### Subgroup analyses

**Table 4** presents the results of subgroup analyses assessing the associations of TYG, TYG-BMI, TYG/HDL, and CHG indices with the risk of carotid atherosclerosis across various demographic and clinical subgroups.

**Table 4:** Subgroup analyses showing the associations between CHG- or TYG-related indices and the risk of carotid atherosclerosis.

Variables		TYG	TYGBMI	TYGHDL	CHG
		OR (95%CI)	OR (95%CI)	OR (95%CI)	OR (95%CI)
Men	Q1	1	1	1	1
	Q2	1.14 (0.95-1.37)	1.03 (0.80-1.25)	1.06 (0.86-1.37)	1.15 (0.91-1.46)
	Q3	1.30 (1.09-1.57)	1.10 (0.88-1.38)	1.22 (0.95-1.57)	1.32 (1.04-1.67)
	Q4	1.18 (0.95-1.46)	1.17 (0.90-1.51)	1.45 (1.11-1.85)	1.48 (1.14-1.91)
Women	Q1	1	1	1	1
	Q2	1.33 (1.02-1.74)	1.14 (0.89-1.47)	1.27 (1.00-1.63)	1.18 (0.91-1.54)
	Q3	1.64 (1.25-2.15)	1.30 (0.97-1.75)	1.89 (1.43-2.51)	1.78 (1.33-2.39)
	Q4	1.52 (1.08-2.14)	1.43 (0.98-2.11)	1.48 (1.03-2.12)	2.19 (1.51-3.18)
Non-HYP	Q1	1	1	1	1
	Q2	1.19 (0.99-1.37)	1.10 (0.92-1.32)	1.27 (1.03-1.56)	1.19 (1.00-1.42)
	Q3	1.33 (1.12-1.59)	1.17 (0.96-1.43)	1.52 (1.25-1.84)	1.49 (1.22-1.81)
	Q4	1.18 (0.92-1.51)	1.21 (0.94-1.57)	1.71 (1.39-2.11)	1.80 (1.42-2.29)
Hyp	Q1	1	1	1	1
	Q2	1.18 (0.83-1.67)	0.80 (0.55-1.15)	1.22 (0.94-1.57)	1.14 (0.87-1.49)
	Q3	1.26 (0.90-1.75)	0.92 (0.64-1.32)	1.59 (1.15-2.06)	1.53 (1.14-2.05)
	Q4	1.27 (0.89-1.80)	0.96 (0.65-1.43)	1.78 (1.29-2.46)	1.95 (1.38-2.75)
LowLDL	Q1	1	1	1	1
	Q2	1.26 (1.03-1.53)	1.16 (0.94-1.44)	1.20 (0.97-1.49)	1.29 (1.06-1.57)
	Q3	1.14 (1.17-1.73)	1.37 (1.09-1.72)	1.45 (1.15-1.83)	1.55 (1.23-1.94)
	Q4	1.38 (1.08-1.76)	1.52 (1.16-2.00)	1.75 (1.37-2.22)	2.04 (1.54-2.71)
HighLDL	Q1	1	1	1	1
	Q2	1.09 (0.63-1.43)	1.14 (0.85-1.53)	1.04 (0.78-1.38)	1.01 (0.62-1.62)
	Q3	1.37 (1.04-1.81)	1.05 (0.77-1.43)	1.33 (0.98-1.81)	1.43 (0.89-2.29)
	Q4	1.20 (0.85-1.68)	1.09 (0.74-1.60)	1.16 (0.82-1.65)	1.50 (0.90-2.51)
NonDM	Q1	1	1	1	1
	Q2	1.18 (1.01-1.39)	1.15 (0.96-1.37)	1.20 (1.00-1.43)	1.21 (1.01-1.44)
	Q3	1.41 (1.19-1.66)	1.24 (1.02-1.50)	1.47 (1.22-1.78)	1.55 (1.27-1.89)
	Q4	1.26 (1.01-1.56)	1.31 (1.04-1.65)	1.68 (1.38-2.06)	1.89 (1.46-2.38)

TYG, triglyceride glucose; TYGBMI, TYG*body mass index; TYGHDL, TYG/high-density lipoprotein cholesterol (C); CHG, cholesterol, high-density lipoprotein, and glucose index.

Model 1 was adjusted for age, sex, body mass index (not for TYGBMI), liver function, renal function, diabetes, albumin, uric acid, blood pressure, low-density lipoprotein cholesterol, triglyceride or high-density lipoprotein cholesterol (for TYG indices).

In male subjects, compared with those in Q1, those in higher quartiles generally presented elevated ORs for carotid atherosclerosis. Notably, the Q3 values of the TYG index (OR 1.30, 95% CI: 1.09–1.57) and the CHG index (OR 1.32, 95% CI: 1.04–1.67) reached statistical significance. Similarly, the Q4 values of the TYGHDL (OR 1.45, 95% CI: 1.11–1.85) and CHG (OR 1.48, 95% CI: 1.14–1.91) indices also presented increased risk estimates. Among female participants, the associations were more pronounced. Compared with those in Q1, the TYG index demonstrated ORs of 1.33 (95% CI: 1.02-1.74) in Q2, 1.64 (95% CI: 1.25-2.15) in Q3, and 1.52 (95% CI: 1.08-2.14) in Q4. The TYG/HDL and CHG indices also reflected similar trends, with Q3 and Q4 showing ORs of 1.89 (95% CI: 1.43-2.51) and 1.48 (95% CI: 1.03-2.12) for TYG/HDL and 1.78 (95% CI: 1.33-2.39) and 2.19 (95% CI: 1.51-3.18) for CHG, respectively.

Stratified analyses based on hypertension status revealed that in participants without hypertension (Non-HYP), particularly TYG/HDL and CHG, increasing quartiles of the indices were significantly associated with increased risk. For example, Q4 of the CHG index was associated with an OR of 1.80 (95% CI: 1.42–2.29). Similar trends were observed in the hypertensive population.

Additional subgroup analyses by low/high LDL cholesterol and diabetic status further substantiated these associations. In the low-LDL-C subgroup, all indices demonstrated a trend toward increased risk, with Q3 and Q4 of the CHG index exhibiting ORs of 1.55 (95% CI: 1.23–1.94) and 2.04 (95% CI: 1.54–2.71), respectively. Similarly, in nondiabetic subjects, higher quartiles of TYG-related indices and CHG (Q3: OR = 1.55, 95% CI: 1.27–1.89; Q4: OR = 1.89, 95% CI: 1.46–2.38) were significantly associated with carotid atherosclerosis, reinforcing the potential utility of these indices in risk stratification.

A subgroup analysis in subjects younger than 40 years showed that the risk of carotid atherosclerosis increased with the CHG (OR = 3.96, 95%CI:1.01-15.84%) after adjusting for age, sex, body mass index, liver function, renal function, diabetes, albumin, uric acid, blood pressure and high-density lipoprotein cholesterol (for TYG indices). However, no such associations were observed in TYG, TYG-BMI and TYG/HDL.

### ROC analyses

**Figure 4:**
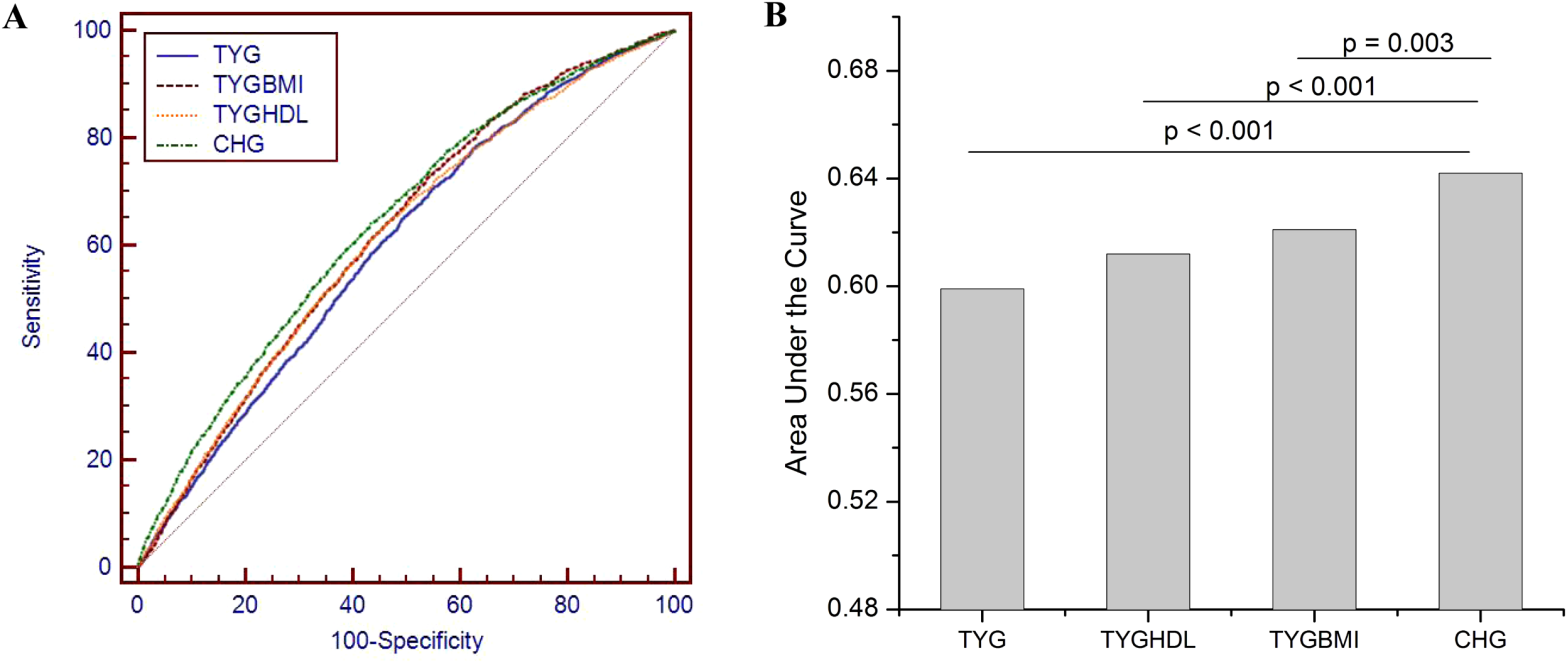
The receiver operating characteristic (ROC) curve demonstrated the performance of the triglyceride glucose (TYG) index, TYG-body mass index (TYGBMI), TYG/high-density lipoprotein cholesterol (TYGHDL) ratio, and cholesterol, high-density lipoprotein, and glucose index (CHG) in identifying carotid atherosclerosis **(A)**. The area under the curve was 0.599 (95% CI: 0.589–0.609) for TYG, 0.620 (95% confidence interval (CI): 0.610–0.631) for TYGBMI, 0.612 (95% CI: 0.602–0.622) for TYGHDL and 0.642 (95% CI: 0.631–0.652) for CHG **(B)**.

**Figure 5:**
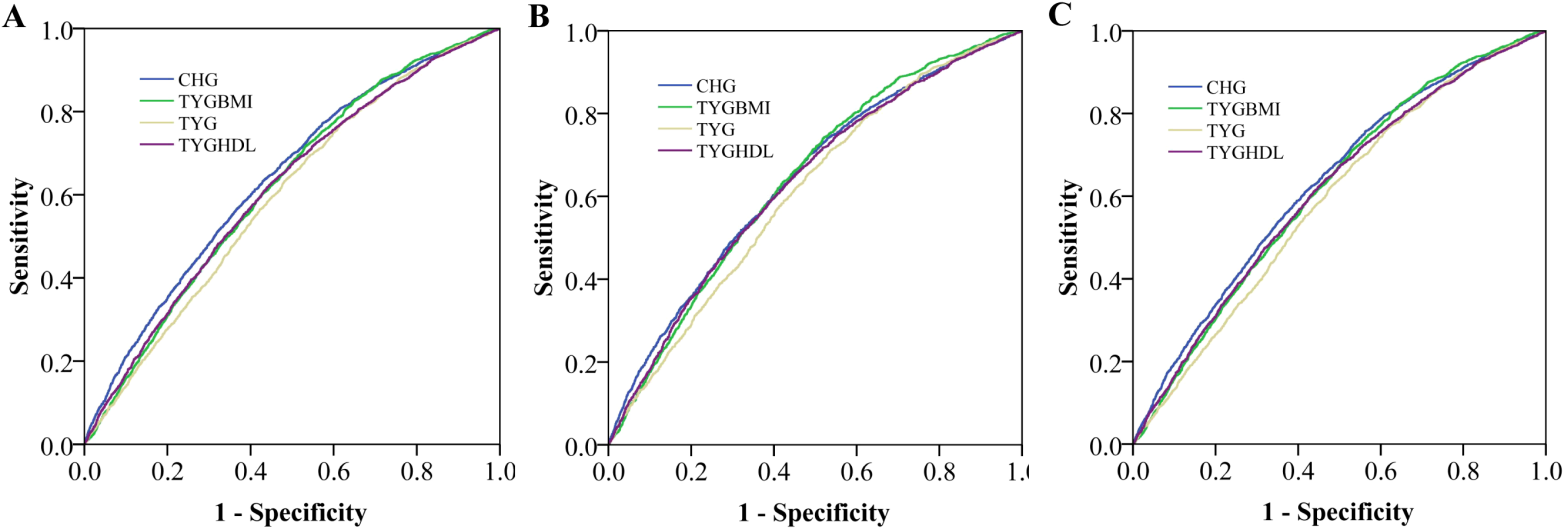
The receiver operating characteristic (ROC) curve demonstrated the performance of the triglyceride glucose (TYG) index, TYG-body mass index (TYGBMI), TYG/high-density lipoprotein cholesterol (TYGHDL) ratio, and cholesterol, high-density lipoprotein, and glucose index (CHG) in identifying carotid atherosclerosis in subjects without hypertension **(A)**, with low LDL-c **(B)**, without diabetes **(C)**. The area under the curve was 0.638 for CHG, 0.618 for TYGBMI, 0.594 for TYG and 0.613 for TYGHDL in subjects without hypertension. The area under the curve was 0.641 for CHG, 0.639 for TYGBMI, 0.610 for TYG and 0.631 for TYGHDL in subjects with low LDL-c. The area under the curve was 0.630 for CHG, 0.615 for TYGBMI, 0.589 for TYG, and 0.610 for TYGHDL in subjects without diabetes.

The ROC curve demonstrated the performance of the TYG index, TYG-BMI, TYG/HDL ratio, and CHG in identifying carotid atherosclerosis (**Figure 4**). The area under the curve was 0.599 (95% CI: 0.589–0.609) for TYG, 0.620 (95% confidence interval (CI): 0.610–0.631) for TYGBMI, 0.612 (95% CI: 0.602–0.622) for TYGHDL and 0.642 (95% CI: 0.631–0.652) for CHG (B). Compared with TYG, TYG-BMI and TYG/HDL, CHG had the greatest ability. Subgroup analyses in subjects without hypertension, without diabetes, and with low levels of LDL-C also revealed that the performance of CHG was greater than that of any other TYG-related indices (**Figure 5**).

## Discussion

As the global burden of CVD continues to rise, the early detection of atherosclerosis, which is the core pathological mechanism underlying CVD, becomes essential for effective prevention and control (23). The risk of factors for CVD in young adults (18–45 years) have been reported, including obesity, hypertension, diabetes, physical activity and lipids levels (18). Carotid atherosclerosis is a key window for assessing subclinical atherosclerosis because of its strong correlation with cardiovascular events (14). The risk factors for atherosclerotic CVD among young adults have garnered increasing attention (24). In this study, we systematically investigated the associations of CHG and TYG-related indices with carotid atherosclerosis for the first time in a young and middle-aged population. Our results revealed that both CHG and TYG-related indices (TYG, TYG-BMI, and TYG/HDL) were significantly and positively correlated with carotid atherosclerosis risk and that the predictive performance of CHG was better than that of TYG indicators in the overall population and in the subgroups without hypertension, diabetes mellitus, and low LDL-C. Our study reported several biomarkers for atherosclerotic CVD among young and middle-aged adults, which is helpful for early CVD management.

The association between the TYG index, a surrogate marker of insulin resistance, and cardiovascular risk has been demonstrated in a variety of populations, (25–27) showing that a higher TYG index was significantly associated with all-cause mortality in young and middle-aged populations (25), and a team also reported that this index, through the cumulative TYG-BMI, was linearly associated with CVD risk (3). A few studies have also shown that TYG is associated with CVD or all-cause mortality in young populations (28, 29). However, few studies have investigated the role of TYG-related indices, such as TYG-BMI and TYG/HDL, in CVD risk in young and middle-aged adults. Moreover, we compared the performances of the TYG and TYG-related indices. Our results showed that TYG-BMI and TYG/HDL had significantly better performance than did TYG.

CHG is another index that can comprehensively reflect the interaction between disorders of glucose and lipid metabolism and IR by integrating cholesterol, HDL-C and blood glucose levels (12). CHG is considered a biomarker of type 2 diabetes (11). Interestingly, a study investigated the predictive ability of CHG and TYG indices in populations aged 45 years and older, and the results revealed that both CHG and TYG indices were associated with CVD risk (12). This study also compared the predictive efficacy of the CHG index and TYG, which have comparable predictive performance (12). Recent two studies also demonstrated that CHG was linked to diabetic retinopathy in diabetic patients, in particularly to proliferative diabetic retinopathy (30, 31). Similarly, an association between CHG and hypertension was also reported (32). Moreover, Tian et al., showed that CHG was not only associated with CVD risk, but also associated with all-cause mortality (33). However, analyses of CHG in middle-aged and young populations are still lacking. To our knowledge, our study may be the first to explore the association between CHG and carotid atherosclerosis in young and middle-aged adults. More importantly, our results revealed that CHG had better performance than did TYG and TYG-related indices. Although the improvement in discriminative ability was modest, given the context of primary prevention and the large population with atherosclerosis, even modest gains in reclassification may carry clinically meaningful implications at the population level. Why CHG had better performance is not clear. One possible reason is that the CHG index contains HDL-C, which is a critical determinant of atherosclerosis.

The role of triglyceride and HDL-c on atherosclerosis has been well studied. Beyond its established role as a surrogate marker of insulin resistance, the CHG index may capture additional triglyceride-driven lipoprotein remodeling that is directly atherogenic. Elevated triglyceride levels are not merely a consequence of insulin resistance but actively contribute to systemic lipoprotein disturbances, including increased very LDL particle number, triglyceride enrichment of LDL particles, elevated remnant cholesterol, and pro-atherogenic modifications of HDL (34). These qualitative lipoprotein alterations promote endothelial dysfunction and arterial wall remodeling through mechanisms partly independent of glycemic status. Triglycerides-rich lipoprotein has been considered as a target for atherosclerosis (35). Therefore, the association between CHG/TyG and carotid atherosclerosis observed in our study may, at least in part, reflect triglyceride-mediated lipoprotein heterogeneity rather than insulin resistance alone.

The incidence of CVD among young adults is receiving increasing attention (18, 36, 37), and early prevention and management of risk factors are vital. There is growing advocacy for prioritizing preventive measures against cardiovascular diseases among younger people (38). CVD risk factors among young or middle-aged adults have also been reported (18, 39, 40). The prevalence of traditional cardiovascular risk factors, including hypertension, hyperlipidemia, and diabetes mellitus, is increasing among younger and middle-aged populations (41). Early screening and effective management of these risk factors constitute critical interventions for mitigating future CVD risks (41). Our study reported several novel factors associated with carotid atherosclerosis in young and middle-aged populations, including TYG-BMI and TYG/HDL, CHG and CHG-TYG scores. Moreover, our results showed that those associations were significant in subjects with low LDL-C. These factors may be valuable in CVD management in young and middle-aged populations. Subgroup analysis showed that CHG was associated with carotid atherosclerosis in subjects younger than 40 years which demonstrated that CHG may be valuable in CVD management in young population.

Metabolic abnormalities in young and middle-aged people are often insidious, and subclinical atherosclerosis may have already progressed before traditional risk factors (e.g., hypertension and diabetes mellitus) met clinical diagnostic criteria. Subgroup analysis in this study revealed an 80% increased risk of CHG in the highest quartile of the nonhypertensive population (OR = 1.80, 95% CI: 1.42-2.29); in the low LDL-C (< 2.6 mmol/L) population, CHG was still significantly associated with risk (Q4 vs. Q1: OR = 2.04, 95% CI: 1.54-2.71), suggesting that CHG may capture metabolic abnormalities not covered by traditional lipid markers, such as IR and HDL functional abnormalities. This finding reinforces the association between “normal range” risk factors and subclinical atherosclerosis in the young population (12) and demonstrates the greater sensitivity of metabolism-related risk in young and middle-aged adults. Therefore, the introduction of novel metabolic indicators in addition to traditional risk stratification tools, such as the ASCVD risk score, is necessary.

It is important to recognize that CHG and TYG-derived indices may reflect distinct underlying pathophysiological processes across different subgroups, particularly among young individuals with early-stage vascular injury. In this population, similar CHG and TYG values may arise from insulin resistance-dominant pathways, triglyceride-rich lipoprotein remodeling, or a combination of both, each with potentially different implications for vascular risk. This heterogeneity is well illustrated by genetic evidence: in patients with heterozygous familial hypercholesterolemia carrying certain LDLR mutations, insulin resistance can emerge independently of LDL-C burden, reflecting broader metabolic vulnerability rather than direct lipid causality (42, 43). Conversely, in other contexts, triglyceride-driven lipoprotein particle modifications may drive atherogenesis even in the absence of overt insulin resistance. Therefore, in young adults with subclinical atherosclerosis, interpreting CHG/TYG associations not as a uniform marker of insulin resistance, but as a composite indicator of cardiometabolic stress, may offer greater conceptual clarity. Future studies integrating advanced lipoprotein profiling (e.g., NMR) and direct insulin sensitivity measurements are warranted to disentangle these distinct metabolic phenotypes and to inform more personalized preventive strategies.

This study has several advantages. 1) This study may be the first to show the association between CHG and carotid atherosclerosis in young and middle-aged adults. 2) We compared the predictive efficacy of CHG and TYG-related indicators for the first time in a young and middle-aged population, confirming the robustness of CHG in a population with different baseline characteristics. 3) We revealed the predictive value of CHG in a population without traditional clinical diseases, providing a new stratification tool for CVD primary prevention. Our study also has limitations. First, the cross-sectional design could not establish causality and requires prospective or longitudinal cohort validation. Second, only carotid intima–media thickness and plaque density were used as surrogate endpoints in this study, and clinical cardiovascular events were not included. Third, we only showed the association between continuous data of CHG and carotid atherosclerosis because the case number of carotid atherosclerosis in young adults (< 40 years) was relatively small. Fourth, future studies may further explore the pathophysiologic mechanisms of CHG, such as its associations with vascular endothelial function and inflammatory factors, and validate its predictive value in a younger population. In addition, our population was a Chinese population. The generalizability of the study results should be validated in other populations. Finally, we did not show an association between CHG- or TYG-related indices and other CVDs, such as coronary diseases and stroke.

In conclusion, the present study confirms that both CHG and TYG-related indices are high-quality noninvasive metabolic markers for early atherosclerosis detection in young and middle-aged populations, with the superior predictive efficacy of CHG, which is particularly valuable for risk stratification in primary prevention. Its integration with traditional risk assessment tools can enhance CVD prevention strategies, but further mechanistic studies and prospective follow-up are needed to improve prospective cohort studies to further clarify the value of CHG in the management and clinical application of cardiovascular risk.

## Data Availability

The original contributions presented in the study are included in the article/supplementary material. Further inquiries can be directed to the corresponding authors.
